# Giving Away a Piece of Yourself

**DOI:** 10.34067/KID.0000000976

**Published:** 2025-10-30

**Authors:** Richard Wenzel

**Keywords:** kidney transplantation, transplant nephrectomy

My wife is, at this very moment, saving my life! Strictly speaking, she will save a stranger's life; once her donor nephrectomy surgery finishes, her kidney will be whisked to the local airport for a multihour flight to a west coast city. Shortly thereafter, her invaluable organ will be implanted, providing this stranger with something that they have likely lost: hope. Hope to escape dialysis' stranglehold. Hope to freely engage in life's marvels. Hope for the future. Stranger, please studiously safeguard my brave wife's treasured gift.

Her altruistic act has an additional reward and is the reason why she underwent a surgery that offers her no tangible health benefits but does offer morbidity and mortality risks; I now qualify to promptly receive a living donor kidney when I require a transplant. At some point in the future, I will be the stranger, wondering about the altruistic person to whom I am indebted for my preemptive transplant.

I was diagnosed with kidney disease during childhood, half a century ago. Thankfully, I have lived life on my own terms, without limitations, but all good things must end and a renal day of reckoning awaits me. I wish that my shrunken (as confirmed by magnetic resonance imaging) kidneys' innate function will sustain me long enough for xenotransplantation or an artificial kidney to become reality, sparing me from immunosuppression's bondage. Yet, I am keenly aware that life's wishes often do not come true.

Not quite a decade ago, my GFR hit the milestone of 20 ml/min, prompting my first visit to a transplant center. At the end of the visit, I was left with this primary question: was anyone that I know willing to donate? At that time, I believed that numerous potential donors existed: siblings, cousins, long-time friends, perhaps even a few coworkers, and, of course, my wife. Since my transplant was not imminent, I did not widely publicize my predicament, rather I sought donors slowly, *via* somber one-to-one conversations at opportune moments. I often began these conversations by pointing out that I have been a registered bone marrow donor for over three decades, hoping that my longstanding willingness to donate *something* might somehow soften my kidney request.

Yet my conversations were repeatedly fruitless, and possible donors dwindled. Most were unwilling to undergo surgery that required weeks of recovery and risked (however small) impairing their long-term capacity to care for their family. Some people cited their own health issues (previously unknown to me) that likely disqualified them. Despite my best efforts toward empathetic conversations, a few people seemed uncomfortable with my request; I apologized and did not press them further.

Asking someone for a kidney lays bare an austere reality: you may have a “good” relationship with someone, but that relationship has limits. Roughly 90,000 individuals currently need a transplant, yet only about 7000 live donors come forth annually; I am one of undoubtedly thousands that have been humbled by relationships' limits. I now regard my wife's decision with an even greater gratitude; the cosmos granted me—of all people—with a donor! Today, I am plotting how to help raise awareness about kidney donations, something I admittedly should have started doing long ago.

I often try to forget my illness, but incursions continually remind me of my kidneys' current peril; my monthly serum laboratory test results arrive to my email with a “High Importance” warning, a consequence of them being significantly abnormal. These emails annoy me; abnormal laboratory results *are* and have seemingly *always been* my life's normal.

I recall inadvertently overhearing a distressing comment not intended for my 10-year-old ears; a physician told my mother that I would not live past the age of 20 years old. Ha! I proved him wrong! Whether his comment arose from that era's medical knowledge limitations, incompetence, or some other reason is unknown. His was the first, but certainly not the last, medical misstep that I would stumble upon, whether as a pediatric patient or years later while working as a front-line clinician myself.

About a decade ago, I sought specialized patient safety training in response to the myriads of things I continually saw go wrong. I viewed the seminal *To Error Is Human* report, published a quarter century ago, as my call-to-action.^1^ Since that time, systematic improvements to patient safety have been, at best, modest.^2^ My own efforts—conversations with colleagues, serving on safety committees, pilot projects—have similarly yielded modest results, at best. As a patient and as a clinician, I view health care through the lens of safety, process improvement, and reliability. Too often, I am troubled by what I see. Alas, the experiences with my wife's surgery were consistent with my entrenched apprehensions.

Recovering in the hospital the day after surgery, somehow my wife's nurse was under the impression that her kidney had been transplanted into me. The nurse was amazed—indeed, flabbergasted—that 1 day after surgery I showed no visible distress and was otherwise functional, attending to my wife's needs. In this nurse's defense, she was temporarily working for the transplant unit and was unfamiliar with asynchronous kidney donations; I provided her with a brief tutorial. This nurse's misunderstanding was innocuous, even humorous, but other events were not.

A week before surgery, hospital staff instructed us to arrive at 4am on the day of surgery. We balked at this early hour, and a few days later, a different staff member relented, saying we could arrive at 4:45am. We did so, only to be told by a security guard—the only person in the hospital's giant entrance lobby at that hour—that Patient Registration does not open until 5am daily. So, we waited, alone, outside, frustrated at this needless 15-minute delay but relieved that we did not arrive at 4am as originally instructed. This experience typifies the torrent of vexing (and often avoidable) blunders, big, and small, that permeate modern health care.

The worst event occurred immediately after surgery, when the surgeon gave me a report. Thankfully, the surgical procedure itself occurred without complications. However, several tubes of blood drawn pre-op (that were to travel with the kidney) had not been labeled and now could not be properly identified. Out of my view, a time-consuming scramble was underway to, first, find suitable substitute tubes and, second, redraw blood from an intravenous access site that, of course, was not reliably functioning. Unfortunately, the deadline for the kidney to board an airplane at the local airport rapidly approached. The surgeon grumbled several less-than-flattering words about the nursing staff. As a medical professional, I knew that his comments were not constructive. Yet as a donor's spouse, I shared his ire. I worryingly wondered whether the currently lacking perfusion kidney might end-up going to waste, doomed by missed flight ischemia syndrome. Helpless, all I could do was await further information.

My wife was discharged yesterday. Here at home, she is sore and does not feel her best but is on-track to a full recovery, nevertheless. I hold her arm as a precaution during short walks outside; we occasionally converse with concerned neighbors. I dispense her pain medications, a task for which I am exceptionally qualified. And I just try to be supportive.

I harbor a solemn, sometimes shamelessly obvious spousal pride. Still, how do I properly and fully honor her selfless act on my behalf? I have resolved to spend the coming years bringing that question's answer to fruition. Right now, I am mixing the ingredients for lemon ricotta pancakes, her favorite. However, I need to pause for a minute. To regain my composure. Lest my tears drip down into the pancake batter.

Addendum—the surgeon ultimately confirmed that, notwithstanding some frantic moments, the kidney arrived on-time and was successfully transplanted. The kidney and the stranger are doing well and are expected to be discharged soon.

## Supplementary Material
